# Supplementary material for: Large-scale manipulation of promoter DNA methylation reveals context-specific transcriptional responses and stability
Source: Genome Biol. 2022 Jul 26;23:163. doi: 10.1186/s13059-022-02728-5 (PMC9316731; doi:10.1186/s13059-022-02728-5)
Supplement: Supplementary file 1 — Additional file 1: Figure S1. The Dox inducible ZF-D3A system. Figure S2. ZF-D3A ChIP-seq replicates show equiparable positional enrichments. Figure S3. Nucleosome position dictates mCH deposition on closed and open chromatin. Figure S4. ZF-D3A-wt expression does not lead to a global reduction of transcriptional activity. Figure S5. ZF binding and DMR induction led to diverse transcriptional responses. Figure S6. Characterisation of promoter DMRs. Figure S7. Examples of non-responsive promoter-DMRs. Figure S8. Differential TF-footprinting at promoter-DMRs. Figure S9. Alternative TSS usage upon Dox induction. Figure S10. Differential ATAC-seq peaks. Figure S11. Differential methylation retention at promoters and distal regulatory elements. Figure S12. Prolonged Dox induction does not increase methylation gain. Figure S13. Epigenomic data overview. Table S1. Global WGBS stats for all samples. Table S2. List of promoters with DMRs and transcriptional change. Table S3. Alternative TSS in noDox vs Dox comparison. Table S4. Alternative TSS in noDox-mut vs Dox-mut comparison. [file 13059_2022_2728_MOESM1_ESM.pdf]

Large-scale manipulation of promoter DNA methylation reveals context-specific transcriptional responses and stability

Alex de Mendoza A, Trung Viet Nguyen, Ethan Ford, Daniel Poppe, Sam Buckberry, Jahnvi Pflueger, Matthew R. Grimmer, Sabine Stolzenburg, Ozren Bogdanovic, Alicia Oshlack, Peggy J. Farnham, Pilar Blancafort, Ryan Lister

Correspondence to: Ryan Lister (ryan.lister@uwa.edu.au), Alex de Mendoza (a.demendozasoler@qmul.ac.uk)

Additional File 1:
Figures S1 to S13.
Table legends S1 to S4.

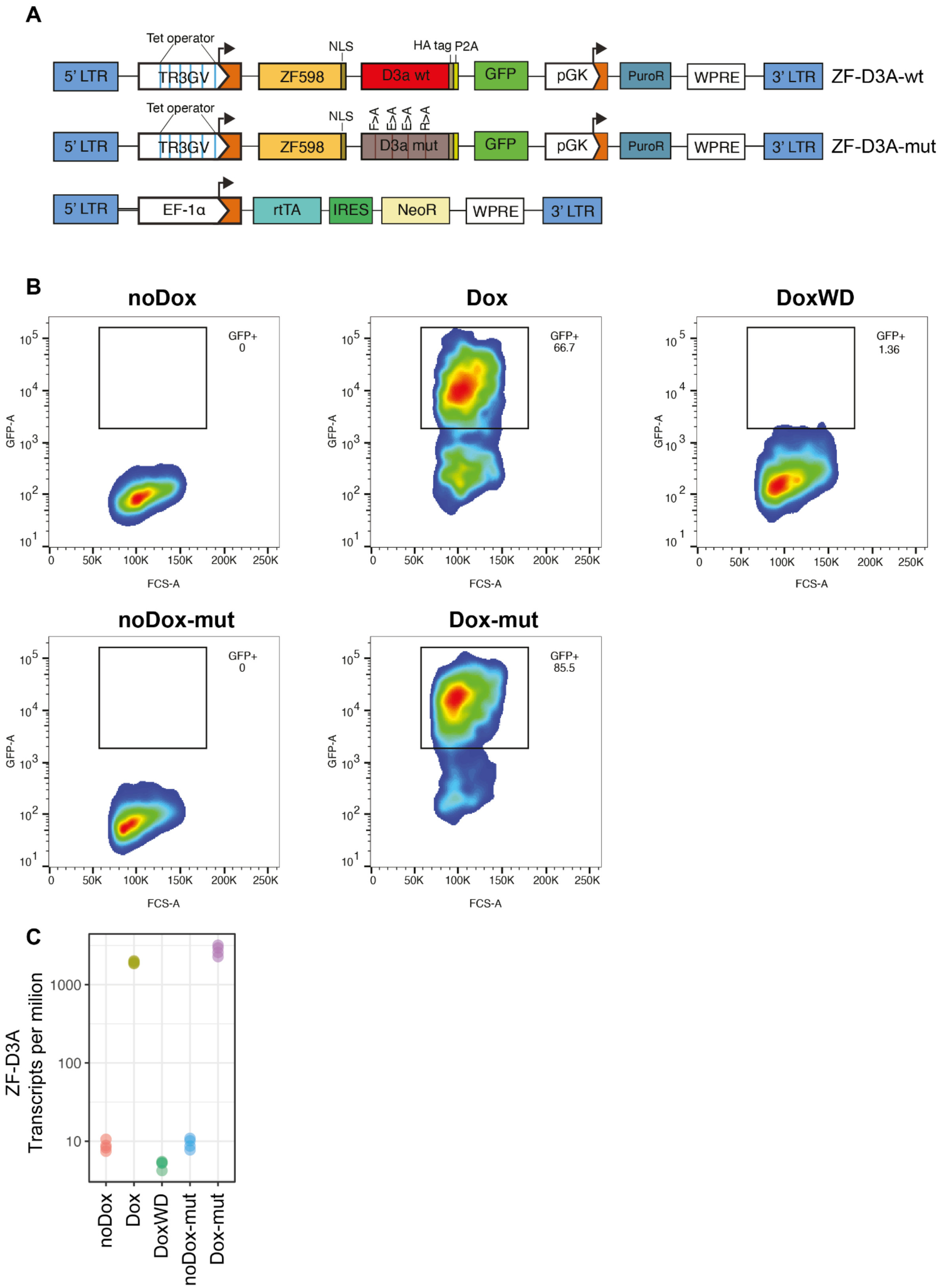

Fig. S1. The Dox inducible ZF-D3A system. A Schematic of the lentiviral constructs used to generate the Dox-inducible MCF7 cell lines. The transactivator rtTA (reverse tetracycline-

controlled transactivator) is constitutively expressed under the control of the EF-1α promoter. In the presence of Dox, rtTA binds TRE3G promoters and activates the expression of ZF fusion constructs. An additional ZF construct and cell-line with mCherry fused to the zinc finger instead of D3A was also generated. **B** FACS profiles showing the proportion of GFP positive cells upon Dox induction in ZF-D3A-wt and ZF-D3A-mut samples. Upon 7 days of Dox withdrawal (DoxWD), GFP signal in ZF-D3A-wt was significantly reduced. The x-axis represents forward scatter area (FCS-A), while the y-axis shows GFP intensity (GFP-A) in log scale. **C** Expression levels of ZF-D3A construct (wildtype and mutant) across samples calculated with Kallisto. This is quantified exclusively using the ZF sequence avoiding the DNMT3A catalytic domain, as it would be confused with the endogenous DNMT3A. The ZF-D3A-wt transcript is the top 67 highest transcribed (out of 22,786 with TPM >1), whereas ZF-D3A-mut transcript is the top 33 highest transcribed (out of 22,913 with TPM > 1).

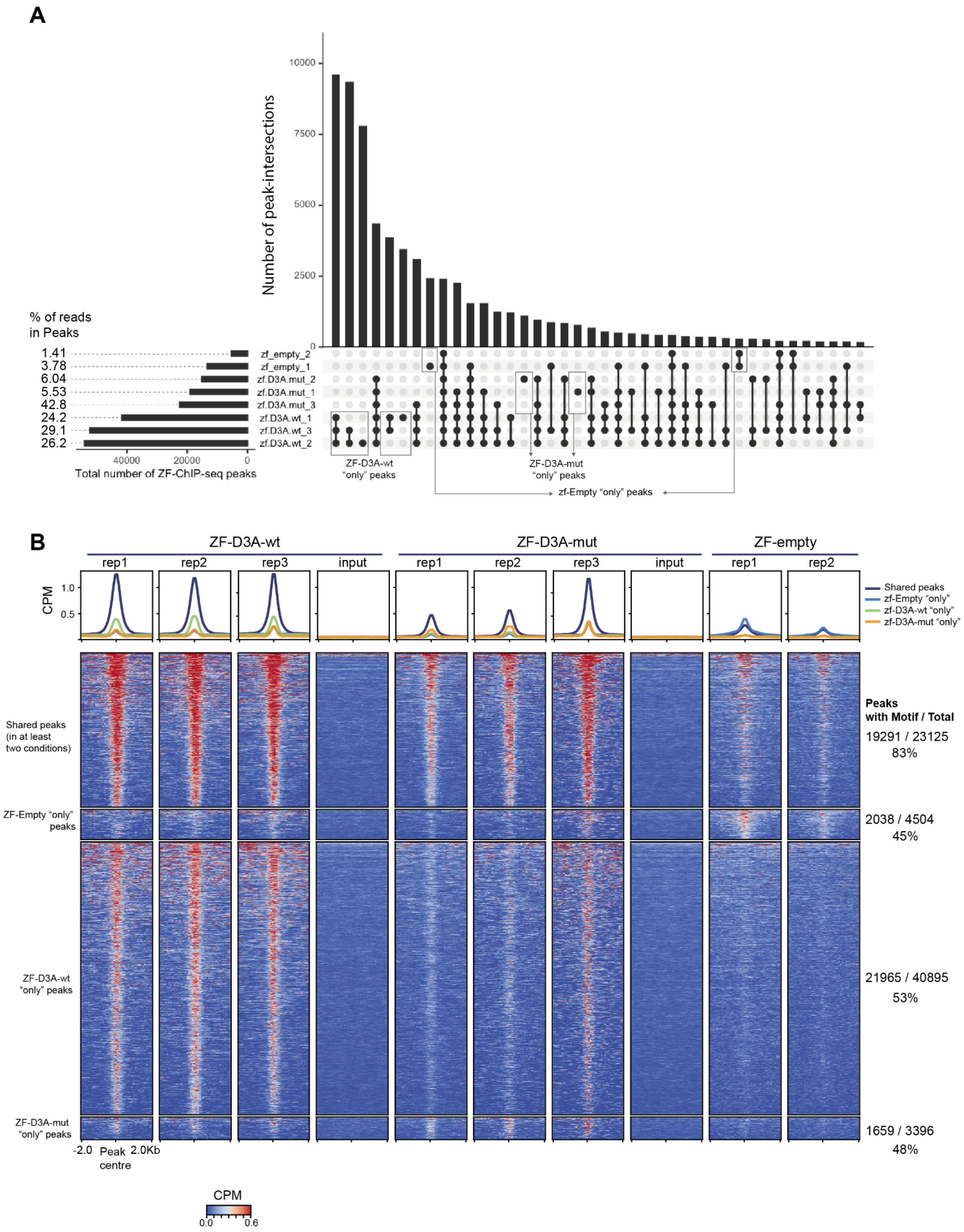

Fig. S2. ZF-D3A ChIP-seq replicates show equiparable positional enrichments. A Intersection between MACS2 ChIP-seq peaks across ZF ChIP-seq samples. Highlighted groups of overlaps are divided in three categories (unique to ZF-D3A-wt, unique to ZF-D3A-mut, or

unique to ZF-empty) that are used in panel B. ZF-empty data comes from a MCF-7 line generated with the same zinc finger without any domain fusion obtained from a previous study[1]. Percentage of reads in peaks was obtained for each sample using the sample-specific MACS2 narrowpeak file. **B** Heatmap depicting raw ChIP-seq coverage on each ZF sample. Read coverage is shown as Counts Per Million (CPM), and input is not subtracted from ChIP data, but shown independently for each cell line. The 4 clusters are defined per overlaps shown in panel A, in which the first represent any peak that overlaps a peak in another condition (e.g. a peak in ZF-D3A-wt_rep1 peak that is also found in ZF-D3A-mut_rep2 will be shown in this cluster). Proportion of peaks overlapping the motif defined in Figure 2a was computed using the scanMotifGenomeWide tool in HOMER2.

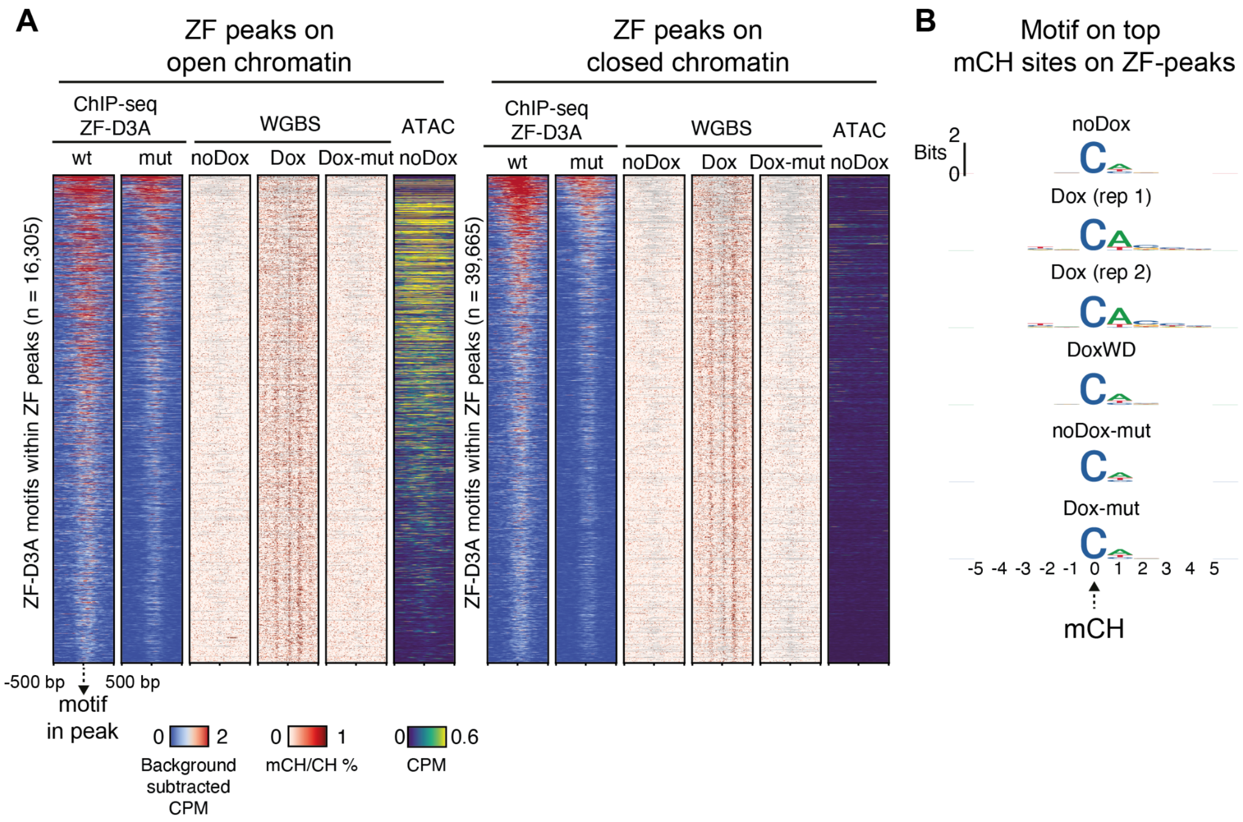

Fig. S3. Nucleosome position dictates mCH deposition on closed and open chromatin. A Heatmaps showing the ChIP-seq signal, mCH %, and ATAC signal on ZF-D3A-wt peaks centered on ZF-D3A motifs. Each ZF-D3A-wt peak can harbour more than one ZF-D3A motif. The motifs are classified according to the intersection with ATAC-seq peaks on the noDox stage. **B** Motif logo on the neighbouring regions flanking top methylated mCH sites on ZF-peaks.

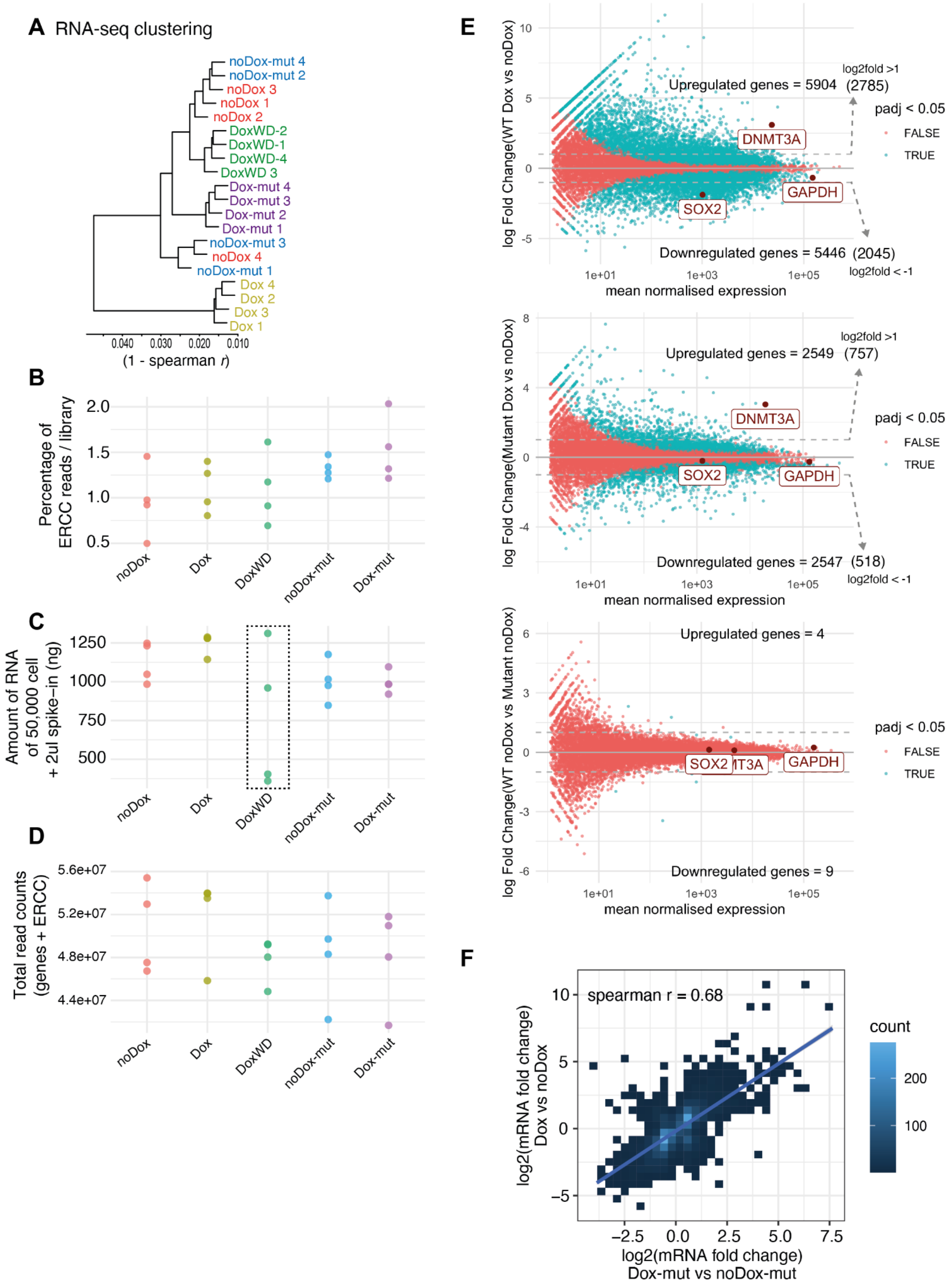
A RNA-seq clustering
noDox-mut 4
noDox-mut 2
noDox 3
noDox 1
noDox 2
DoxWD-2
DoxWD-1
DoxWD-4
DoxWD 3
Dox-mut 4
Dox-mut 3
Dox-mut 2
Dox-mut 1
noDox-mut 3
noDox 4
noDox-mut 1
Dox 4
Dox 2
Dox 3
Dox 1
(1 - spearman r)
B
Percentage of ERCC reads / library
C
Amount of RNA of 50,000 cell + 2ul spike-in (ng)
D
Total read counts (genes + ERCC)
noDox
Dox
DoxWD
noDox-mut
Dox-mut
E
log Fold Change(WT Dox vs noDox)
Upregulated genes = 5904 (2785)
log2fold >1
Downregulated genes = 5446 (2045)
log2fold < -1
padj < 0.05
FALSE
TRUE
DNMT3A
SOX2
GAPDH
mean normalised expression
log Fold Change(Mutant Dox vs noDox)
Upregulated genes = 2549 (757)
Downregulated genes = 2547 (518)
log Fold Change(WT noDox vs Mutant noDox)
Upregulated genes = 4
Downregulated genes = 9
F
spearman r = 0.68
count
log2(mRNA fold change) Dox vs noDox
log2(mRNA fold change) Dox-mut vs noDox-mut

Fig. S4. ZF-D3A-wt expression does not lead to a global reduction of transcriptional activity. A Clustering of the RNA-seq replicates by spearman correlation. **B** Percentage of reads mapping to ERCC spike-ins in each replicate for each stage. noDox and Dox do not show significant differences (Wilcoxon rank test $p > 0.05$). **C** Total amount of RNA measured with the Qubit RNA BR kit after RNA purification. For all samples but for DoxWD (7 days), 50k cells were used, spiked with 2 μL of ERCC Mix1 or Mix 2 (1:100 stock dilution) before RNA purification. For DoxWD, fewer cells could be obtained (13734, 50000, 42252, 13734), but the ratio of spike-in was modified to maintain the same proportion as for the other samples. **D** Total read count for each sample based on reads mapping to genes and ERCC mRNAs. **E** MA-plots showing the distribution of transcriptional changes per gene in relationship to transcriptional level as per DEseq2 normalised values. Dashed lines depict a fold change of 2 (log2 transformed). *DNMT3A* is a positive control for overexpression, since the sequence of ZF-D3A is identical to the endogenous *DNMT3A* gene, and *SOX2* is the originally targeted gene with the predicted best match for the ZF-D3A. **F** Comparison of the log fold changes of genes between Dox versus noDox for ZF-D3A-wt and Dox versus noDox for ZF-D3A-mut.

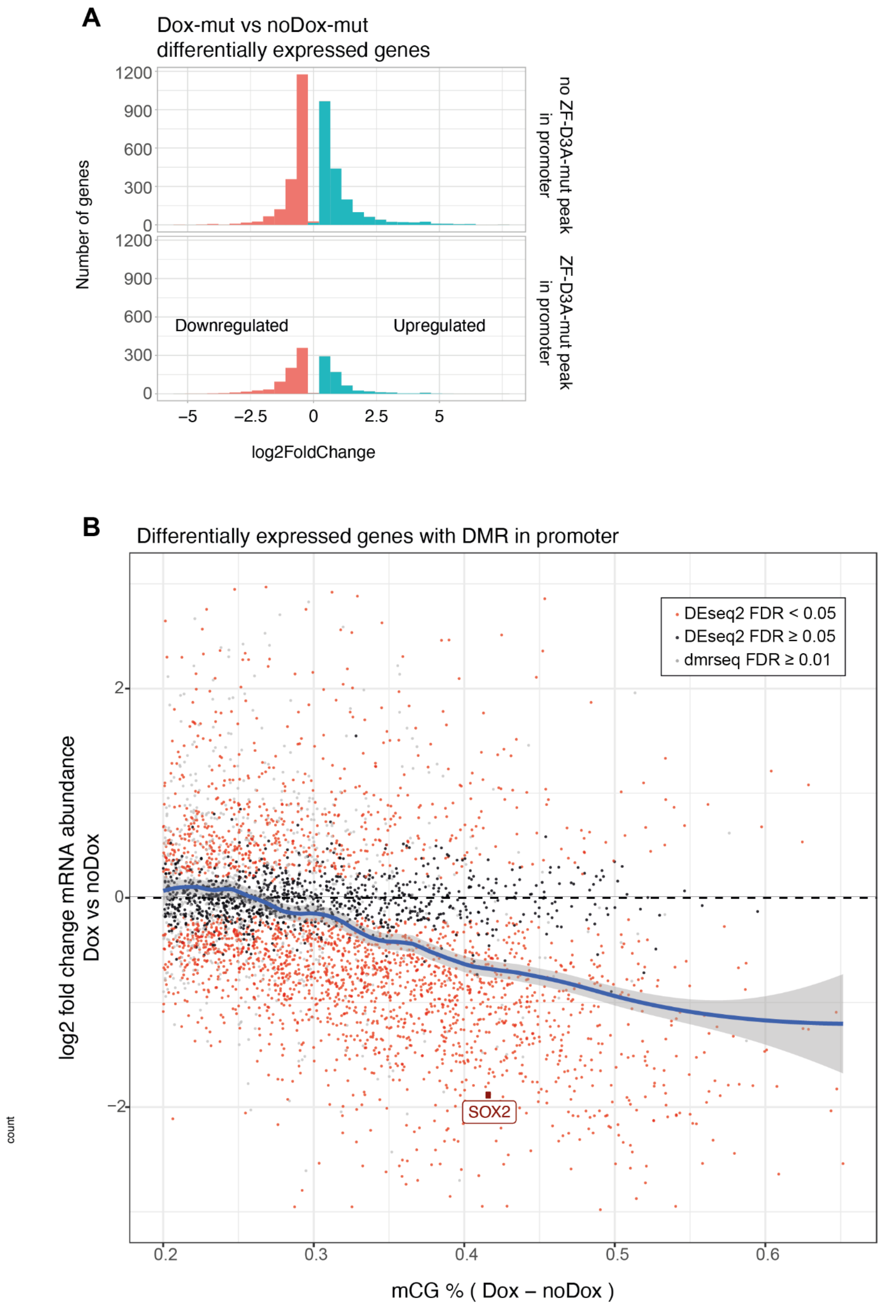

Fig. S5. ZF binding and DMR induction led to diverse transcriptional responses. A Distribution of log fold transcriptional levels between Dox-mut and noDox-mut for differentially expressed genes divided by the presence of a ZF-D3A-mut peak on the promoter of the gene. **B** Scatter plot of the DMR methylation gain (as defined by weighted methylation difference on the DMR, unlike **Fig. 3D**), versus the fold change in mRNA abundance of promoter-DMR associated genes, between noDox and Dox. Point color indicates the gene differential expression significance: red indicates FDR <0.05, black indicates FDR >0.05 (DEseq2). Trend line (blue) was fitted using a Local Polynomial Regression. Depicted genes are not differentially expressed in the ZF-D3A-mut.

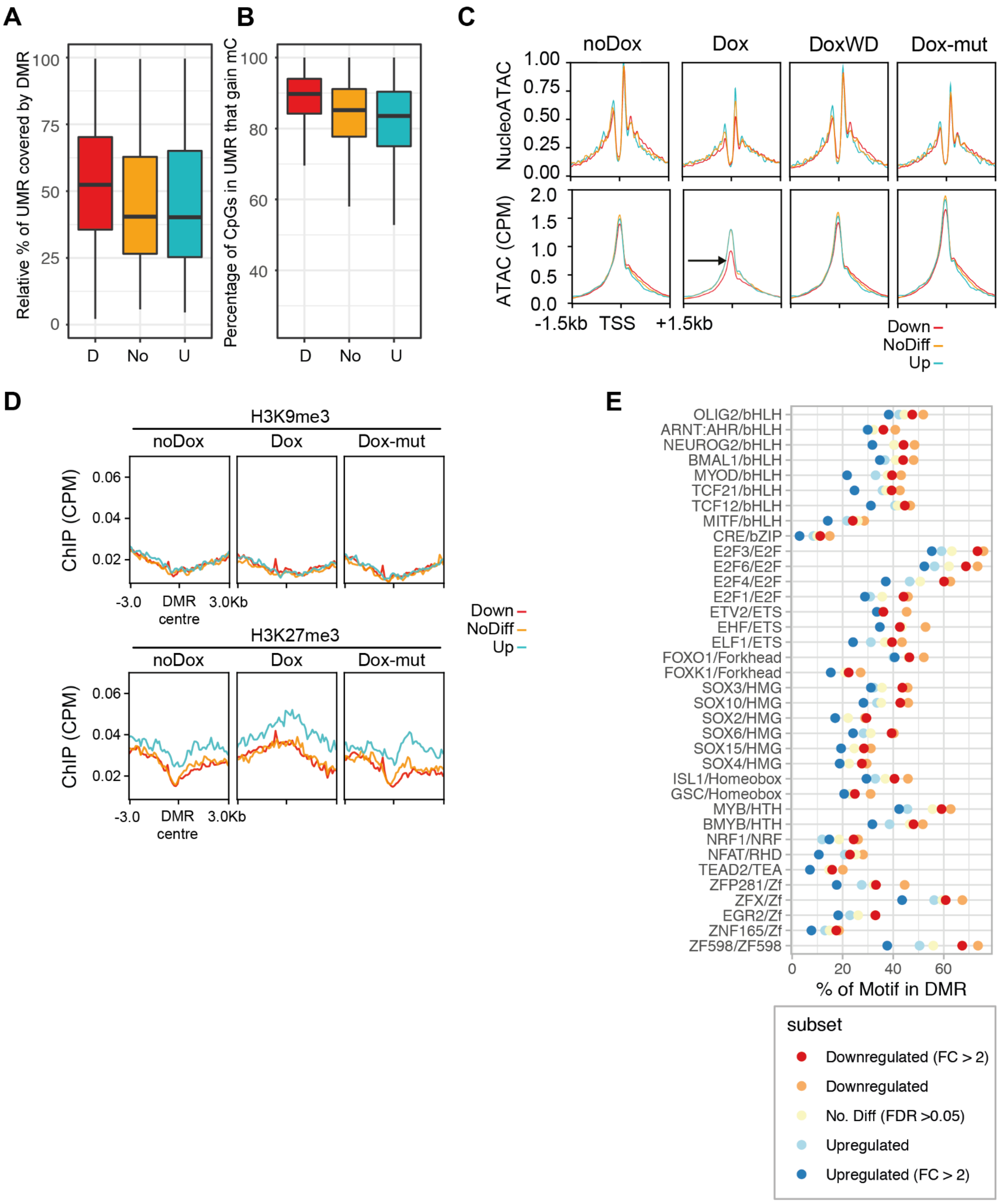

Fig. S6. Characterisation of promoter DMRs. A Distribution of percentage of the promoter UMR covered by the DMR. **B** Proportion of CpGs on the promoter UMR that gain methylation upon Dox induction. Boxplot centre lines are medians, box limits are quartiles 1 (Q1) and 3 (Q3), whiskers are 1.5 × interquartile range (IQR), and points are outliers. **C** Nucleosome spanning and raw ATAC-seq signal around the TSS of genes with promoter DMRs divided by transcriptional

response upon Dox induction. **D** ChIP-seq signal of H3K9me3 and H3K27me3 on promoter-DMRs, categorized by the transcriptional response of the associated gene upon Dox induction. **E** Percentage of DMRs that harbor a TF binding motif divided by transcriptional response to Dox induction.

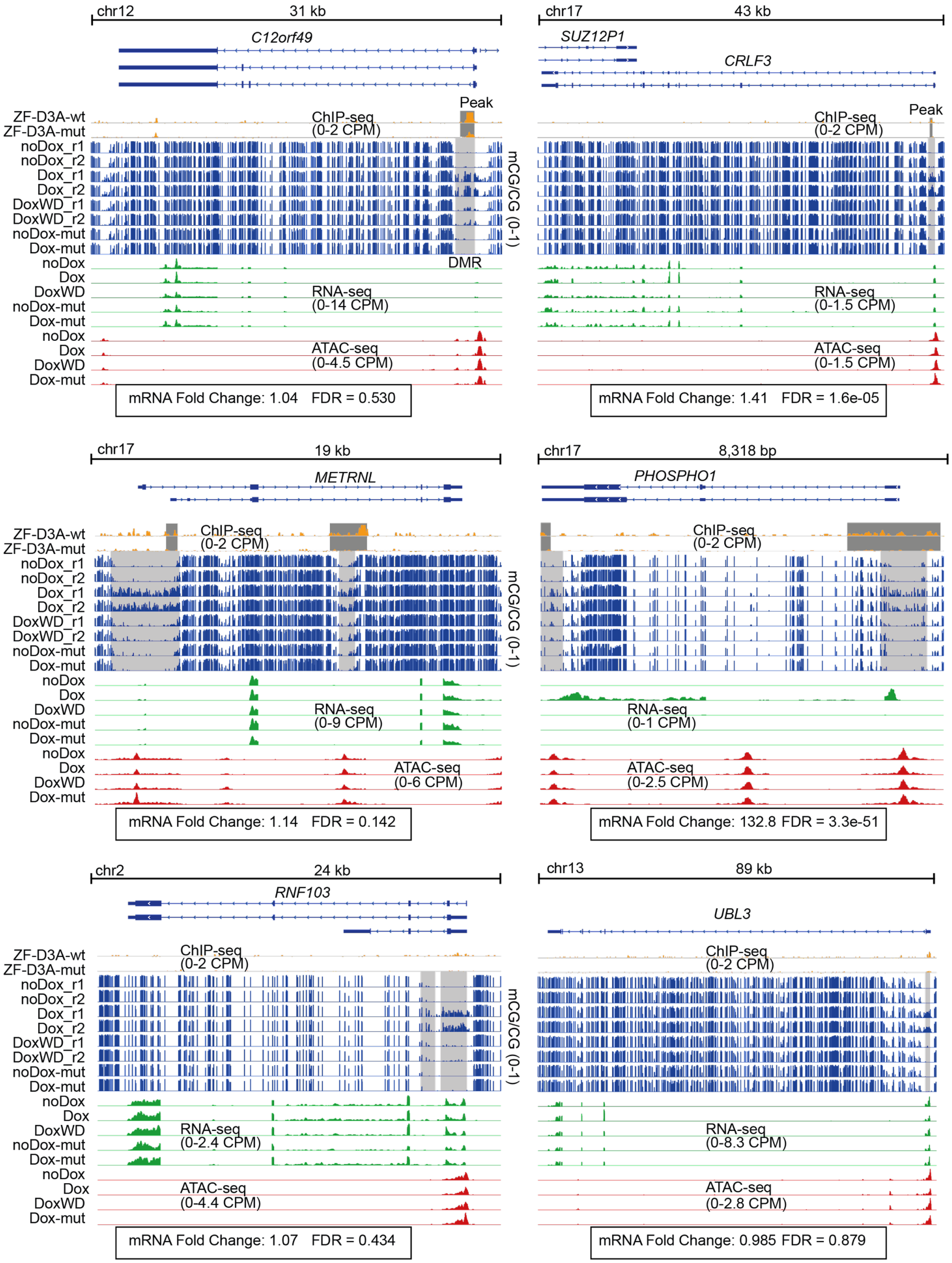

Fig. S7. Examples of non-responsive promoter-DMRs. Genome browser snapshots showing

various promoter-DMRs that are not associated with gene repression. Dark grey shading on the ZF-D3A ChIP-seq tracks represents the peaks called by MACS2, and the lighter grey shading on the WGBS tracks represents the DMRs called with dmrseq. mRNA Fold Change indicates the transcriptional change in the Dox versus noDox comparison, where 1 indicates no change, and >1 indicates upregulation. The FDR is the DESeq2 p.adjusted value.

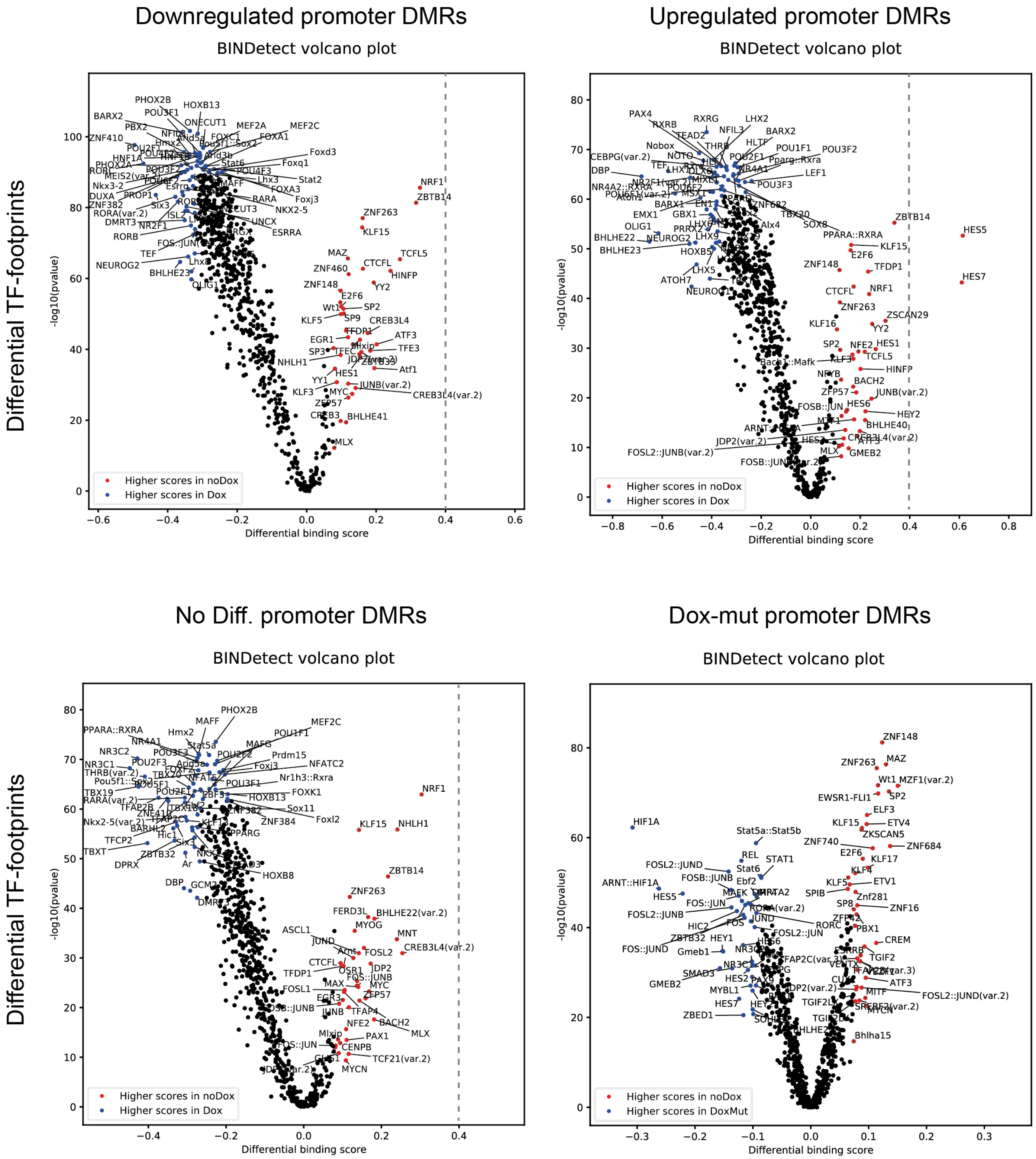

Fig. S8. Differential TF-footprinting at promoter-DMRs. Volcano plots showing the differential footprints assigned to Jaspar core-vertebrate TF motifs across the distinct promoter-DMRs. Right dots are statistically significant footprints calculated by TOBIAS that are depleted upon Dox induction, and blue dots indicate footprints that are gained. The bottom right plot shows TF footprints on all promoter-DMRs that show differences between Dox-mut and noDox, showing a different set of motifs than those depleted in Dox promoter-DMRs. This indicates that ZF binding is not responsible for the loss of these footprints.

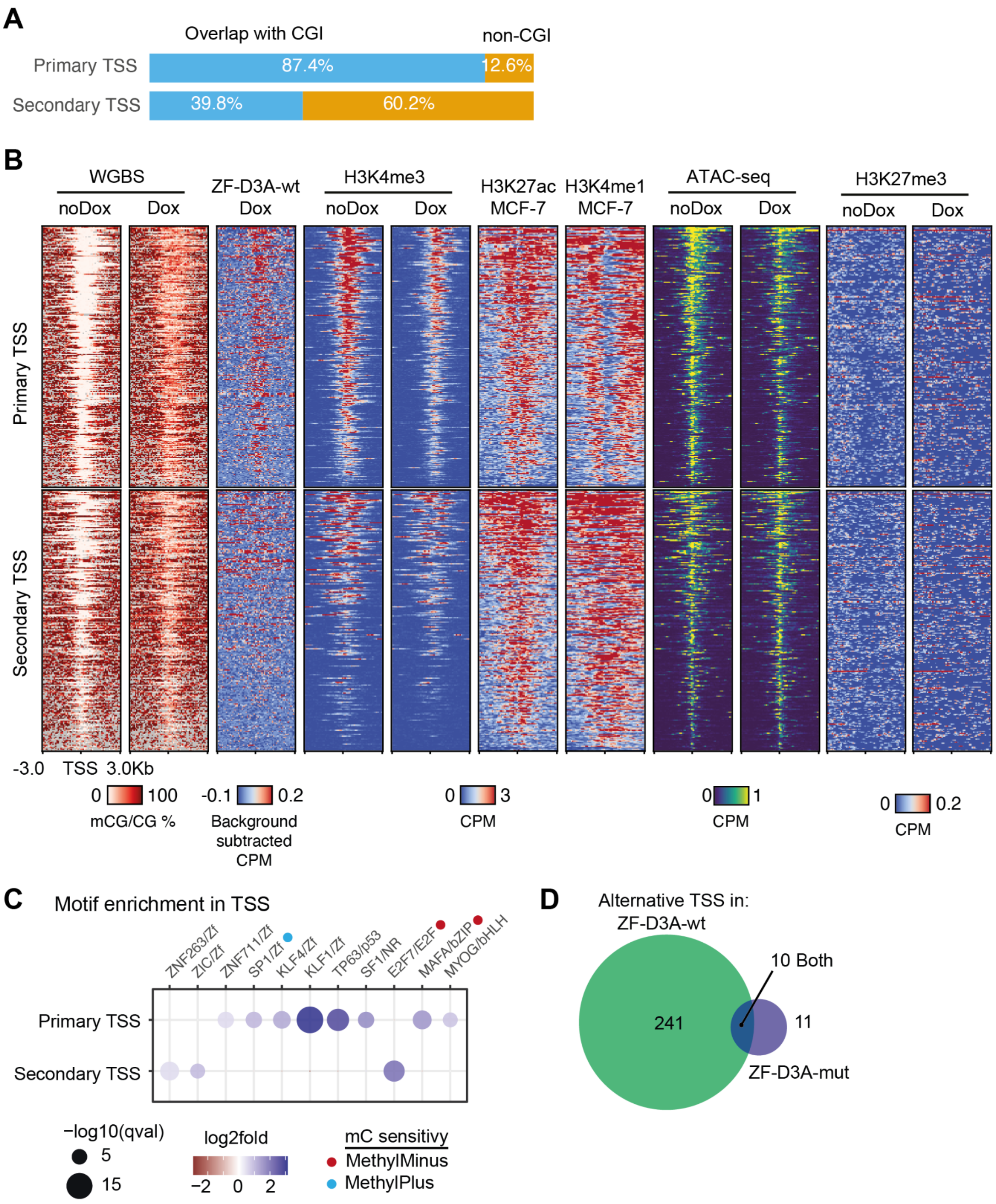

Fig. S9. Alternative TSS usage upon Dox induction. A Proportion of primary and secondary TSS that overlap with CpG Islands. **B** Heatmap showing methylation, ZF-D3A-wt, H3K4me3, H3K27ac, H3K4me1, H3K27me3 signal, and chromatin accessibility on primary and secondary

TSS. H3K27ac and H3K4me1 bigwigs mapped to hg19 were downloaded from the ENCODE database. **C** TF binding motif enrichments comparing primary TSS versus secondary TSS. Many of the ZF are enriched in CpGs, which reflects the higher CpG densities of primary TSS. **D** Overlap between alternative TSS usage in ZF-D3A-wt noDox vs Dox and ZF-D3A-mut noDox vs Dox comparisons. Of the primary TSS that are alternatively transcribed in ZF-D3A-mut, only 8 intersect with ZF-D3A peaks, while none of the secondary TSS do.

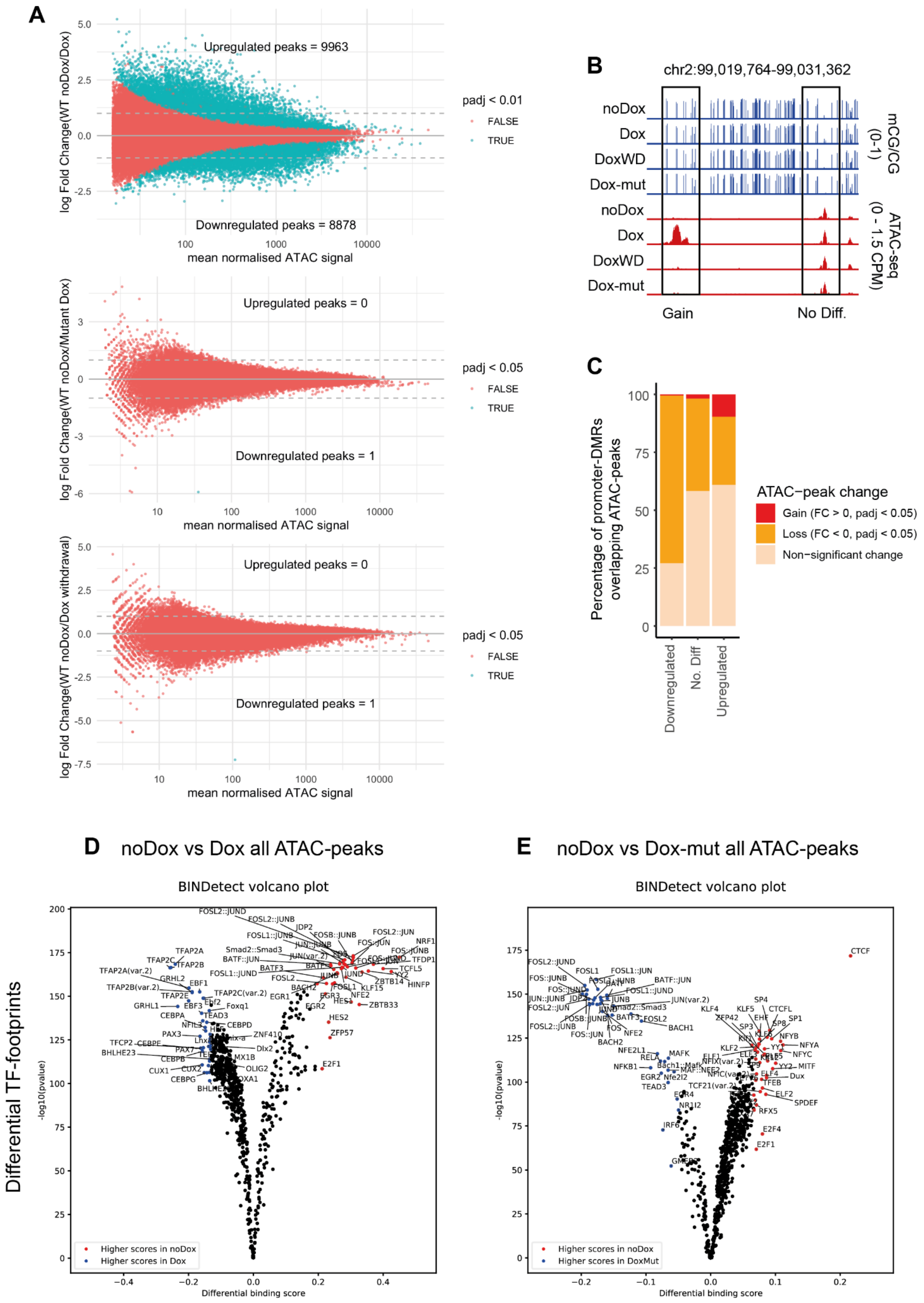
A
Upregulated peaks = 9963
Downregulated peaks = 8878
log Fold Change(WT noDox/Dox)
mean normalised ATAC signal
padj < 0.01
FALSE
TRUE
Upregulated peaks = 0
Downregulated peaks = 1
log Fold Change(WT noDox/Mutant Dox)
mean normalised ATAC signal
padj < 0.05
FALSE
TRUE
Upregulated peaks = 0
Downregulated peaks = 1
log Fold Change(WT noDox/Dox withdrawal)
mean normalised ATAC signal
padj < 0.05
FALSE
TRUE
B
chr2:99,019,764-99,031,362
noDox
Dox
DoxWD
Dox-mut
mCG/CG (0-1)
ATAC-seq (0 - 1.5 CPM)
Gain
No Diff.
C
Percentage of promoter-DMRs overlapping ATAC-peaks
ATAC−peak change
Gain (FC > 0, padj < 0.05)
Loss (FC < 0, padj < 0.05)
Non−significant change
Downregulated
No. Diff
Upregulated
D
noDox vs Dox all ATAC-peaks
BINDetect volcano plot
Differential TF-footprints
-log10(pvalue)
Differential binding score
Higher scores in noDox
Higher scores in Dox
E
noDox vs Dox-mut all ATAC-peaks
BINDetect volcano plot
-log10(pvalue)
Differential binding score
Higher scores in noDox
Higher scores in DoxMut

Fig. S10. Differential ATAC-seq peaks. **A** MA-plots showing the differential binding across ATAC-peaks obtained using DEseq2. Only the noDox vs Dox comparison identifies differential peaks, whereas Dox-mut or DoxWD do not show differential peaks. **B** Genome browser snapshot of a Dox-specific ATAC peak gained on a methylated region. **C** Intersection between promoter-DMRs classified by transcriptional change of the associated gene upon Dox induction and ATAC-peaks. Downregulated genes with promoter-DMRs overlap mostly with ATAC-peaks that lose accessibility, whereas upregulated genes with promoter-DMRs have the highest proportion of peaks that gain accessibility upon Dox induction. **D** Differential TF footprint calculated for the intersection of all ATAC-peaks in any stage. In red, footprints depleted upon Dox induction, in blue footprints enriched upon Dox induction. **E** Differential TF footprint on the same set of ATAC-seq peaks but using Dox-mut data. Many of the motifs that are depleted in Dox versus noDox, are enriched in Dox-mut (FOS, JUN).

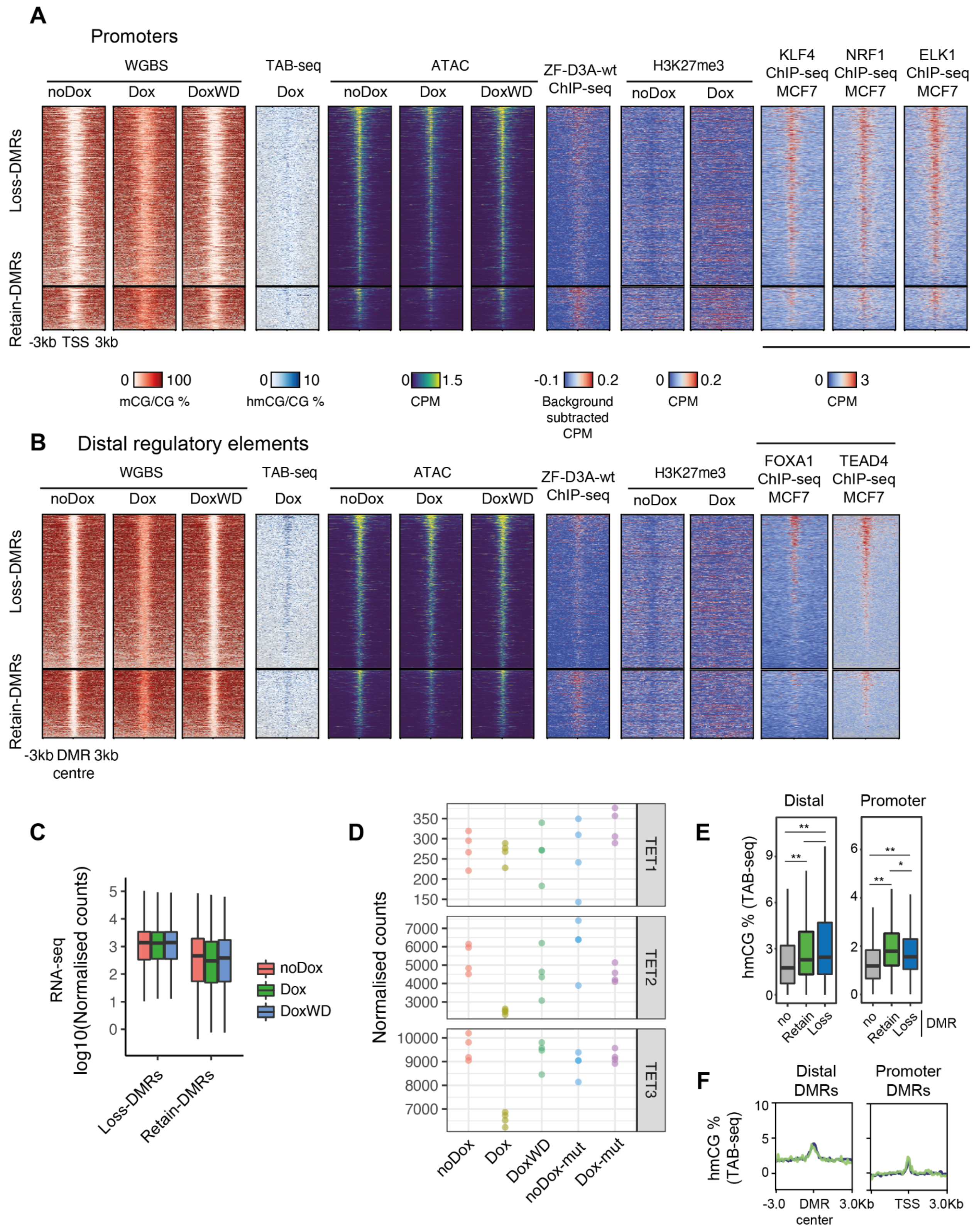

Fig. S11. Differential methylation retention at promoters and distal regulatory elements. Heatmap showing methylation and hydroxymethylation levels, chromatin accessibility, and TF binding on DMRs that retain or lose DMR upon Dox withdrawal, divided in **A** promoters and **B**

distal regulatory elements. **C** Distribution of gene expression levels for genes with promoter-DMRs classified as retain-DMR or loss-DMR. **D** Expression of genes encoding the *TET* enzymes across all conditions, where each dot represents the normalised expression level (DEseq2) in each replicate. *TET2* and *TET3* are downregulated upon Dox induction. **E** Distribution of hydroxymethylcytosine (hmCG) values on promoters or distal regulatory elements. Those regions that do not overlap DMRs are displayed in grey, those that overlap Retain-DMRs are shown in green, and those that overlap Loss-DMRs shown in blue. An asterisk indicates a significant one-sided t-test ($p < 0.0001$), two asterisks indicate a lower p-value. **F** Average % hmCG on DMRs and TSS of genes with DMRs. The boxplots centre lines are medians, box limits are quartiles 1 (Q1) and 3 (Q3), whiskers are 1.5 × interquartile range (IQR), and points are outliers.

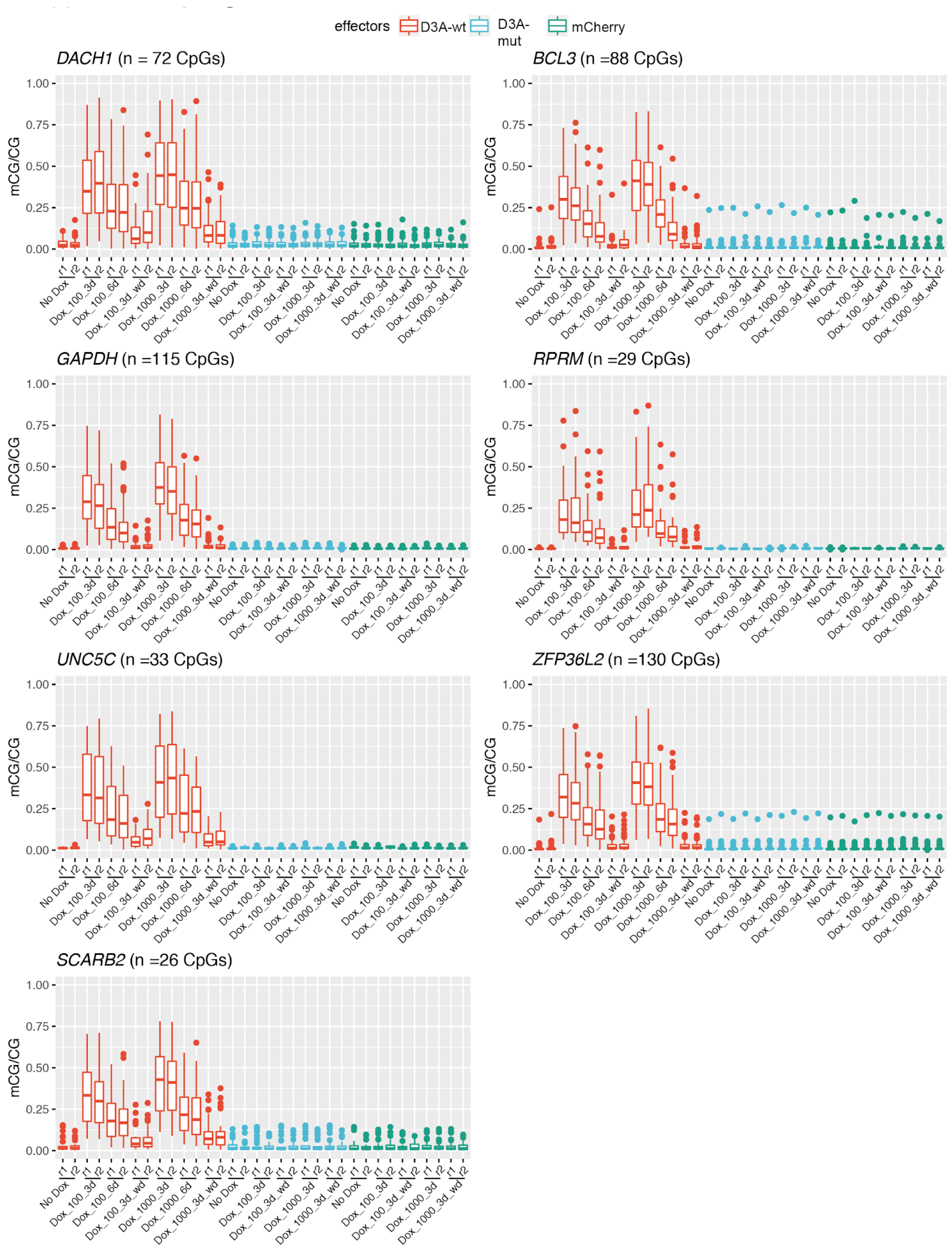

Fig. S12. Prolonged Dox induction does not increase methylation gain. Average CpG methylation levels on seven gene promoters obtained through bisulfite PCR amplicon sequencing

in duplicates. Dox_100 indicates 100 ng/ul of Dox used per induction, whereas Dox_1000 indicates 1000 ng/ul. 3d and 6d indicate 3 days and 6 days of Dox induction, respectively. Colour coded as per cell line construct. Red indicates ZF-D3A-wt, blue indicates ZF-D3A-mut, and green is ZF-mCherry.

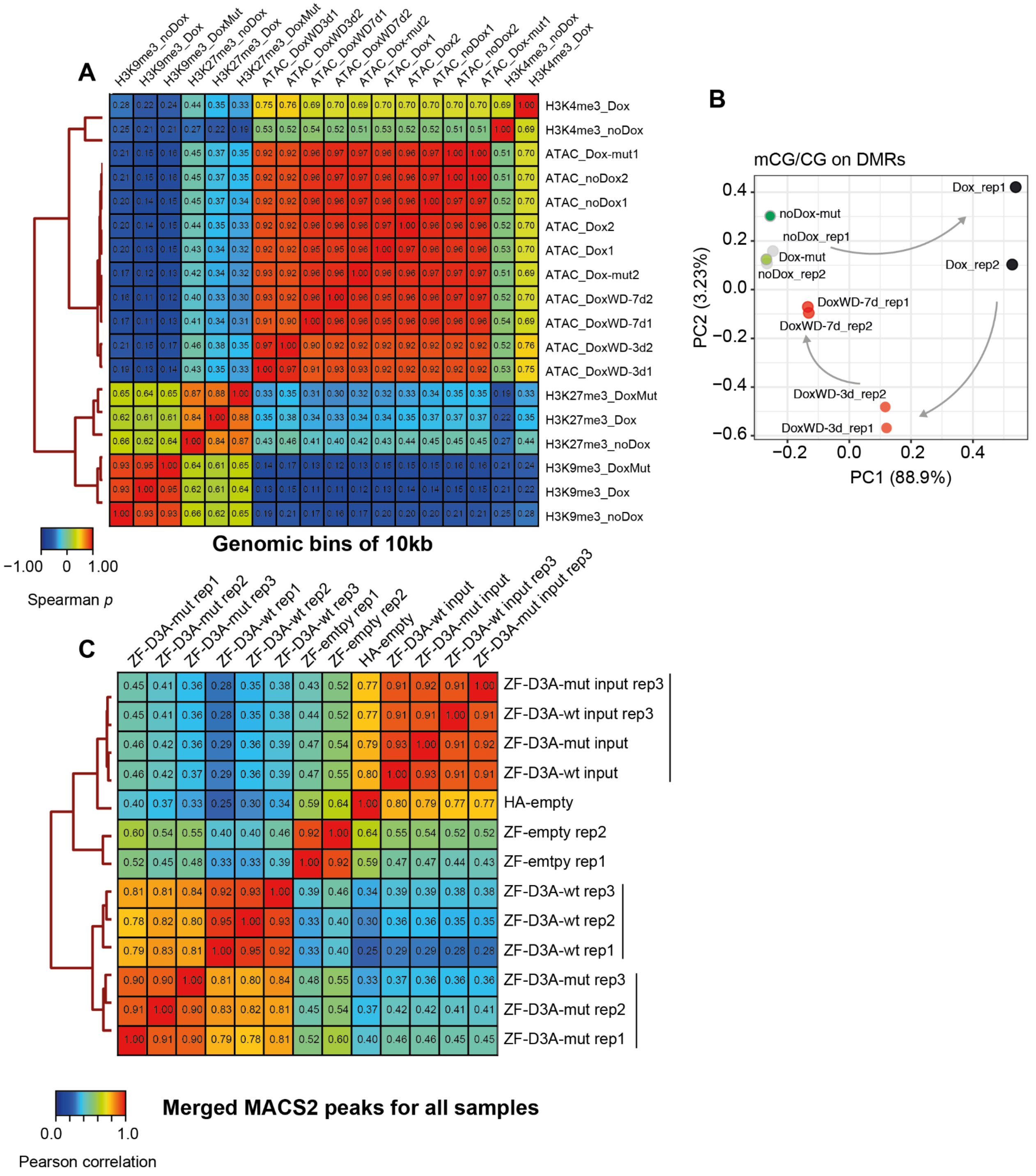

Fig. S13. Epigenomic data overview. A Correlation clustering between H3K4me3/H3K9me3/H3K27me3 ChIP-seq and ATAC-seq samples using the mean coverage values in 10 kb windows across the genome. **B** Principal component analysis of WGBS libraries classified as mCG level on DMRs defined in noDox vs Dox samples. **C** Correlation clustering between Zinc Finger ChIP-seq and input tracks using the mean coverage values in the merged list of MACS2 peaks for all samples.

Table S1. Global WGBS stats for all samples. Global methylation levels at CpG sites on the human genome and the unmethylated lambda spike in control.

Table S2. List of promoters with DMRs and transcriptional change. Coordinates of the DMRs (FDR < 0.01) intersecting promoters, with dmrseq statistics. Associated gene RNA-seq values as computed per DEseq2, for the noDox vs Dox comparison. Classified by category of transcriptional change and per intersection with ZF-D3A-wt. Those genes that are also differentially expressed in the ZF-D3A-mut noDox-mut vs Dox-mut are also shown.

Table S3. Alternative TSS in noDox vs Dox comparison. SEASTAR alternative first exon coordinates with the differential Percent Splice Inclusion (PSI) values.

Table S4. Alternative TSS in noDox-mut vs Dox-mut comparison. SEASTAR alternative first exon coordinates with the differential Percent Splice Inclusion (PSI) values.
